# Telocytes and Other Interstitial Cells 2.0: From Structure to Function

**DOI:** 10.3390/ijms232416221

**Published:** 2022-12-19

**Authors:** Sanda Maria Cretoiu

**Affiliations:** Department of Morphological Sciences, Cell and Molecular Biology and Histology Discipline, Carol Davila University of Medicine and Pharmacy, 050474 Bucharest, Romania; sanda.cretoiu@umfcd.ro

Interstitial cells are often seen as those cells that fill the space between parenchymal cells, responsible for fulfilling the function of an organ. These cells are organized in 3D structures made with the help of elements from the extracellular matrix, such as collagen fibers, elastin, reticulin, and glycoprotein molecules (such as GAGs, proteoglycans, and adhesive molecules). In the cell biology of interstitial cells, a new heterogenous distinctive population was described in recent years under the name of “telocytes” [1]. In the diverse landscape of cellular components found in the interstitial space, there are distinct populations that can be grouped according to the functions they perform in: (a) cells with a supporting role, also acting as immune regulators, e.g., fibroblastic reticular cells [2]; (b) cells with a role in the synthesis of extracellular matrix components, e.g., fibroblasts [3]; (c) stem/progenitor cells, e.g., mesenchymal cells [4]; (d) mechanochemical sensors or stem cell nurses, e.g., telocytes [5]; (e) cells with pacemaker properties, e.g., interstitial cells of Cajal [6], to mention but a few principal categories.

This Special Issue, titled “Telocytes and Other Interstitial Cells 2.0: From Structure to Function”, represents the continuation of a previous issue, reflecting the importance of these cells in the field of intestinal cell research [7]. The Special Issue collected two original papers and four reviews on telocytes.

With their original results, Rosa and colleagues were the first to demonstrate that the telocyte network in the dermis of the skin of bleomycin-treated mice is severely compromised [8]. These results correlate with their previous study on the skin of scleroderma patients where they described the damage and loss in the telocyte network in the areas with cutaneous fibrotic disorders [9]. Telocytes from the dermis of bleomicin-treated mice were studied by immunofluorescence and immunoblotting and electron microscopy. The results showed that the majority of telocytes suffered injury and degeneration at the ultrastructural level, while others (few in number) underwent transdifferentiation in myofibroblasts, changing their immunophenotypic characters from CD34+ to α-SMA+ [8].

The in vitro study performed by Kanno et al. was dedicated to aortic valve interstitial cells found in hypoxic culture conditions which were studied for gene expression profiles using RNA sequencing [10]. The authors have provided the first molecular evidence regarding the fact that hypoxia promotes valve interstitial cell differentiation to osteocytes. Although the study has certain limitations, the results were promising regarding the stemness properties of cultivated cells in hypoxic conditions, opening new perspectives for the identification of future therapeutic targets [10].

Telocytes are peculiar cells, as described in 2010 by Professor Popescu’s Romanian team who established the “platinum criteria” for their identification in almost every organ, including the gut [1,11]. Telocytes have clear distinctive immunophenotypical characters, i.e., CD34-positive/c-kit-negative/platelet-derived growth factor receptor α (PDGFRα)-positive characters in the gut, which can be differentiated by the classical interstitial cells of Cajal (ICC), known to be c-kit-positive/CD34-negative/PDGFRα-negative [12]. An interesting review contained in this collection is a paper by Maria Giuliana Vannucchi which tackles the interactions between telocytes and intestinal resident macrophages [13]. There is clear ultrastructural evidence that telocytes and macrophages establish cell-to-cell contacts, but the review clearly makes a distinction between the role of this relationship depending on the localization in all four tunics of the intestinal wall. Moreover, this interesting work proposed a theory about a possible telocyte–macrophage–smooth muscle cell (TC-MC-SMC) circuit and discuss it by comparison with the SIP syncytium (consisting of SMCs/ICC/PDGFRα+ cells) theory proposed by Sanders et al. [13,14].

Telocytes were also described in turtle, mouse, rat, and adult human testes [15,16,17,18]. Regarding this topic, Shin-ichi Abe assembled a valuable review and proposed an in vitro system to examine the functions of telocytes in spermatogenesis [19]. He and his group elaborated and studied the reconstruction of in vitro seminiferous tubule-like structures, in which they dissociated testicular cells deprived of interstitial cells and peritubular cells and concluded that, in the absence of all the above-mentioned components, dysmorphic structures are formed [20]. They decided that all the cell components are indispensable for the reconstruction of seminiferous tubule-like structure and that this model scientist will be able to investigate the role of Leydig cells, as well as interstitial and peritubular cells, such as CD34+PDGFR+ mesenchymal cells (telocytes), immune cells, and endothelial cells, by knocking out some genes at a time [19].

The role of telocytes in inflammatory bowel diseases is explored by Banciu et al. who elaborated a theoretical model based on various mechanisms and hypotheses emitted over time in relation to the functions of telocytes in the interstitial space of different organs [21]. The paper reviews the main knowledge relating to the presence of, and morphological and quantitative changes in, telocytes in the inflammatory diseases of the digestive tract, such as Crohn’s disease, ulcerative colitis, and irritable bowel syndrome. The hypothesis is based on pH changes, evolutionary changes in the intestinal flora in correlation with food variation, as well as mechanical changes (degree of distension) in the intestinal wall in correlation with the functional relationships of the telocytes with other cell types, nerve endings, and blood vessels [21]. The model, although theoretical, can reflect the functional complexity of these poorly understood cells—the telocytes.

The review by Klein et al. discusses the controversial role of telocytes, mainly related to their presence in the structure of the heart and their regenerative potential in tandem with the stem cells [22]. For the second time, the Slovakian group proposed that telocytes should be included in official histological nomenclature *Terminologia Histologica* after 16 years since their first description [23].

The second edition of this Special Issue dedicated to telocytes and interstitial cells aimed to gather and assess current research in the field as a continuation of the first Special issue, with the hope that more and more groups of researchers will join their results to finally discover the functions of these somewhat strange cells.
